# Wear effects between polymethyl methacrylate occlusal splints and opposing dentin surfaces during bruxism mimicking events

**DOI:** 10.1038/s41405-023-00148-6

**Published:** 2023-06-10

**Authors:** Prangtip Potewiratnanond, Cholakorn Ekrojanakul, Tatcharee Harikul, Rapatporn Kositvanich

**Affiliations:** 1 Department of Occlusion, Faculty of Dentistry, Chulalongkorn University, Bangkok, Thailand; 2 Faculty of Dentistry, Chulalongkorn University, Bangkok, Thailand

**Keywords:** Occlusion, Dentine, Oral diseases

## Abstract

**Objectives:**

To compare the surface wear rate between polymethyl methacrylate (PMMA)-based occlusal splints and opposing dentin-exposed teeth in bruxism simulating models.

**Materials and methods:**

PMMA-based occlusal splints and extracted premolars were tested on a chewing stimulator at 30,000 or 60,000 cycles. Dentin wear was measured under a stereomicroscope and PMMA wear was determined with an optical profilometer. In addition, wear surface topography was assessed and quantified by scanning electron microscopy (SEM).

**Results:**

Wear rate of PMMA was significantly greater (11 times) than that of dentin specimens at 60,000 cycles, though these findings were not observed at 30,000 cycles. When comparing wear rates within each group at different duration cycles, PMMA surfaces exhibited an average wear rate 1.4 times higher with high duration cycles, while dentin surfaces displayed a marginal decrease in wear. In SEM micrographs, PMMA surfaces displayed more wear abrasion lines with higher duration cycles. However, dentin surfaces did not exhibit major differences between low and high duration cycles.

**Conclusion:**

Wear rate on PMMA-based occlusal splints remarkably increases upon high chewing cycles mimicking bruxism comparing with the rate on dentin. Hence, it is reasonable for bruxing patients to wear single-arch PMMA-based occlusal splints to protect opposing dentin-exposed teeth.

## Introduction

Aging leads to human tooth wear, one of the major restorative challenges in dentistry. Dental wear builds up through time as a result of different physical and/or chemical insults. Consequently, this wear has an impact on patients’ oral daily routines and their satisfaction in terms of their visual appearance, pain levels, oral comfort, chewing ability and swallowing. Epidemiological studies on tooth wear have been reported in many countries. In the particular case of Thailand, the prevalence of tooth wear within the population age range of 60–74 years is 51.9% for occlusal surface wear and 44.6% for incisal surface wear [1]. In another Asian country, China, a large cross-sectional investigation on the prevalence of tooth wear in several dental surfaces reported that the occlusal and incisal wear are the most prevalent when compared with other surfaces [2].

Occlusal stabilization splints have become one of the major therapies for treating pathological tooth wear caused by bruxism [3]. These splints are commonly used because of their non-invasiveness, relative affordability, and convenience. Further, occlusal splints are believed to prevent tooth wear from clenching during bedtime while acting like a ‘night guard’ [4]. These appliances cover all teeth in one of the dental arches, either maxilla or mandible, preventing these teeth to contact with their opposing counterparts [5].

PMMA is a popular dental resin material since it displays several relevant properties and advantages including high cost-effectiveness, promptly conferring aesthetics features, easy to manipulate, reasonable oral tissue biocompatibility with limited reported allergic reactions among others [6–9]. Currently, PMMA is commonly used for dental prosthetic applications, including the fabrication of occlusal splints. However, occlusal splints are commonly designed to cover only one dental arch, and this raises clinical concerns about the potential wear effects on the opposing natural tooth surfaces that are not covered by the splints. This is especially true in the case of patients with moderate to severe bruxism who are recommended to wear occlusal splints as standard therapy. Such patients typically have dentin-exposed teeth which are more sensitive to surface wear than enamel-exposed ones [10]. Thus, this study aims to determine dentin and PMMA surface wear rates between PMMA-based occlusal splints and dentin-exposed tooth surfaces upon different chewing cycles mimicking bruxism.

## Materials and methods

### Biosafety and ethical guidelines

The study was approved by the Human Research Ethics Committee of the Faculty of Dentistry, Chulalongkorn University, Bangkok, Thailand (approval certificate no. 2020-047). All laboratory experimentations were done under safety standard operating procedures which were approved by the Institutional Biosafety Committee of the Faculty of Dentistry at Chulalongkorn University.

### Natural tooth samples

Twelve extracted premolars (*n* = 12) without any pathological condition, such as caries, enamel hypoplasia, abfraction or any other surface lesion, and without any patient identifiers were collected from private dental clinics in Bangkok, Thailand. Enamel from the occlusal surfaces was removed by a dental bur (Diamond disc, Dianfong, China). Then, occlusal surfaces were grounded by SiC papers of 600 grit (Shanghai abrasive tools, China) to produce a flat dentin surface area in the natural teeth. All tooth samples were stored at room temperature in water before testing.

### Fabrication of heat-cured PMMA occlusal splints

Standard heat-curing powder (Rodex, S.P.D., Italy) was used to fabricate PMMA occlusal splints by performing a standard lost-wax and resin packing technique. The polymer and monomer of the PMMA kit were mixed following the manufacturer instructions. For polymerization, the filled mold was heated to 80 °C for 15 h. Afterwards, all splint samples were finalized with stepwise polishing processes using sandpaper, wet pumice on a brush wheel and tallow on a rag wheel. Every grinding procedure was done in cold water to prevent overheating.

### Material testing outcome measurements

1. Dentin and PMMA wear through a chewing simulator mimicking bruxism
  Specimens were mounted on a chewing and wear simulator (CS-4.4, SD Mechatronik, Feldkirchen-Westerham, Germany) as seen in Fig. 1. Since mechanical properties of dentin vary according to its internal structure as well as external environment [10, 11], wet conditions were created by submerging dentin and PMMA specimens in distilled water to simulate saliva-lubricated oral surfaces (Fig. 1c). Dentin specimens were embedded in a custom-made holder, while circular cylinder-shaped PMMA occlusal splints with 10 mm in diameter and 3 mm height were mounted to antagonize such dentin specimens. A vertical load was applied at 50 N and with 2 mm of horizontal movements at the speed of 1.6 cycles per minute. Cycle frequency was programmed for 1.42 Hz and temperature of the simulator chamber was set at 25 °C [12]. Half of the dentin-exposed tooth specimens (*n* = 6) were tested at 30,000 loading cycles, while the other half at 60,000 loading cycles to resemble different number of bruxism cycles in bruxing patients [13, 14].

**Fig. 1:**
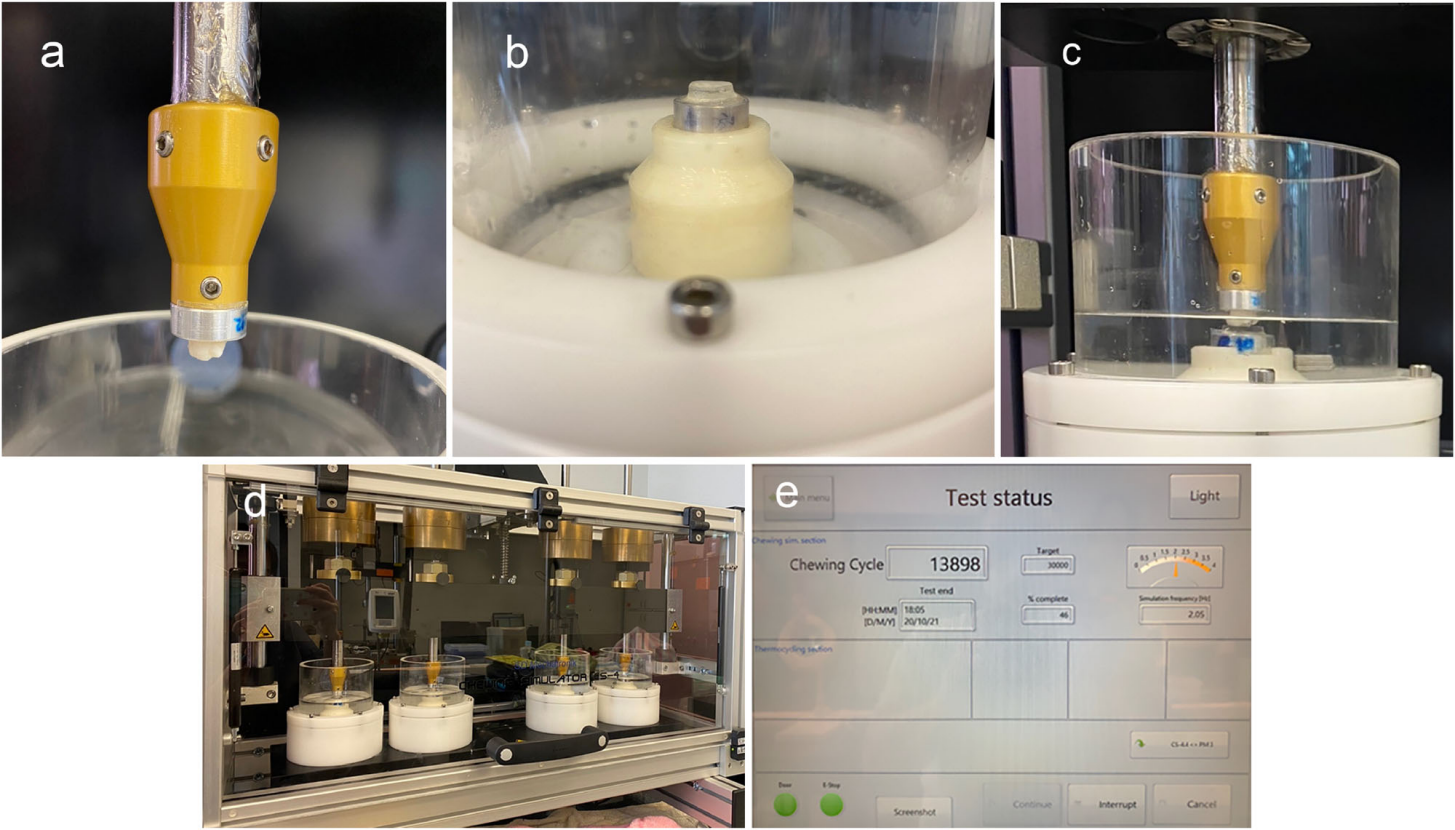
A chewing and wear simulator. Mounted dentin-exposed tooth (**a**) and PMMA splint specimens (**b**) were submerged in water (**c**) and tested on a CS-4.4 SD Mechatronik simulator (**d**) to generate bruxism-like cycles set up by the software as per screen display (**e**).
  Dentin wear was calculated by measuring the tooth height under a stereomicroscope (SZ 61, OLYMPUS, Japan) before and after the bruxing simulation. Furthermore, each dentin-exposed tooth specimen was positioned on a plaster index to maintain the same position, and measurements were taken by the same observer to eliminate inter-observer variability.
  PMMA material wear was scanned as a three-dimensional object and analyzed with a non-contact profilometer (InfiniteFocusSL, Alicona Imaging, Austria).
2. Scanning electron microscopy

After bruxing simulation and wear measurement, all specimens were prepared for taking SEM micrographs (Quanta FEG 250, FEI, United States). Specimens were removed from the wear test holder and mounted in the stub using a conductive adhesive. Afterwards, they were coated with 99.99% gold by smart coater (JFC-1200, JEOL, United States). SEM was set at high voltage and HiVac at vacuum mode. To prevent damage to the PMMA resin surfaces, wear surfaces were imaged up to 1000× as higher magnifications could generate heat from the electron beam.

### Statistical analysis

Wear rate of dentin and PMMA surfaces were compared at 30,000 and 60,000 loading cycles of bruxism. SPSS version 22 (IBM, Armonk, New York, USA) for windows operative system was used to analyze the data with a set up alpha level at 5%. Shapiro–Wilk test was used to confirm normal data distribution. Mean differences between dentin and PMMA groups were compared using Mann–Whitney U test at different bruxism duration cycles. In addition, Wilcoxon Signed Ranks test was used to compare wear rates between 30,000 and 60,000 loading cycles of dentin and PMMA groups.

## Results

Table 1 exhibits the wear of dentin and PMMA at the low and high bruxism mimicking cycles, respectively. At low duration bruxism events (30,000 cycles), no differences in dentin and PMMA surface wear was present (Table 1). Conversely, when high duration bruxism events are mimicked (60,000 cycles), PMMA surfaces displayed 11 times more wear on average than dentin surfaces (*p* = 0.024, Table 1). In addition, when comparing wear rates at different bruxism duration cycles, the wear rate on PMMA surfaces was on average 1.4 times higher with high duration cycles (*p* = 0.053). Though, there was a marginal decrease in wear on dentin surfaces (*p* = 0.106).

**Table 1:** Dentin and PMMA wear (mm) at different bruxism mimicking cycles.

Bruxism cycle	Specimens	Mean ± SD	95% CI				
			Lower bound	Upper bound	Minimum	Maximum	*P* values
30,000	Dentin	0.015 ± 0.013	−0.006	0.036	0.000	0.030	*0.276*
	PMMA	0.023 ± 0.010	0.007	0.038	0.010	0.030	
60,000	Dentin	0.003 ± 0.005	0.002	0.009	0.000	0.010	*0.024*^a^
	PMMA	0.033 ± 0.008	0.025	0.042	0.020	0.040	

*CI* confidence interval, *SD* standard deviation.

^a^Mann–Whitney U Test. Statistical significance was set at *P* < 0.05.

Italic values denote statistically significant *p* values.

**Fig. 2:**
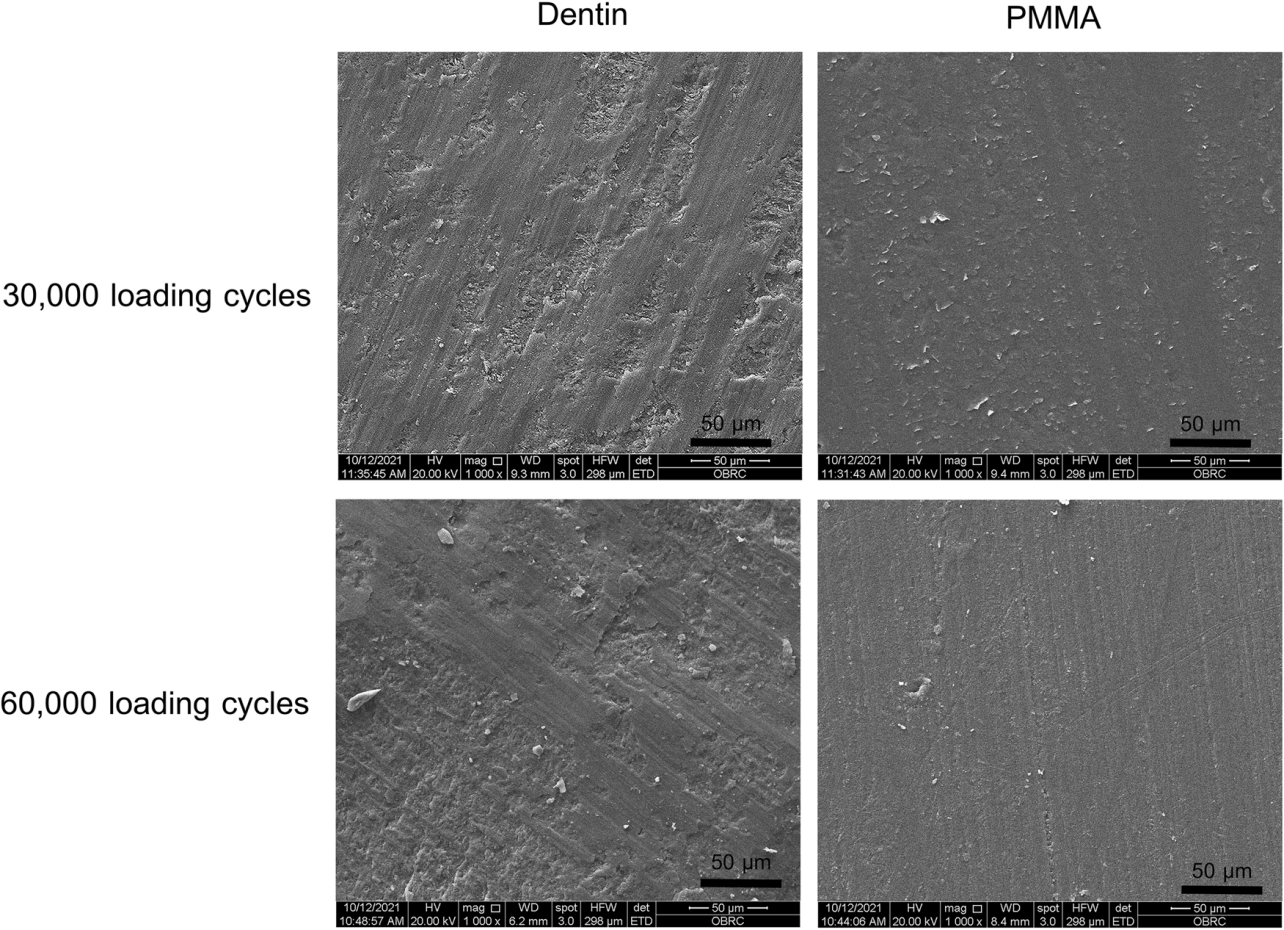
SEM micrographs of surface wear in dentin and PMMA after bruxism simulation events. Magnification: ×1000.

Surface wear appearance and topography in high magnification SEM micrographs are shown in Fig. 2. In dentin surfaces, micrographs exhibited irregular surfaces with a few abrasion lines. Also, such images on dentin surfaces did not display any major differences between 30,000 and 60,000 cycles. On the other hand, SEM micrographs of PMMA surfaces showed greater number of straight lines of smooth surface abrasions upon increasing from 30,000 to 60,000 cycles, mainly in one plane direction. As expected, more inhomogeneity and surface roughness were present in dentin surfaces when compared to the PMMA surfaces.

## Discussion

The fabrication of full coverage PMMA occlusal splints for one of the dental arches is generally recommended for bruxing patients to ameliorate the harmful effects of tooth wear [3]. Several studies reported a greater hardness in enamel structures intrinsic to their large inorganic phase composition [10, 11, 15, 16]. In addition, PMMA material has been shown not to wear down enamel-exposed teeth [17]. Based on these findings, researchers and clinicians deemed reasonable to wear only one occlusal splint either covering the maxillary or mandibular dental arches if only enamel is exposed. Though, one would expect such wear to be different if patients have dentin-exposed teeth since dentin has significantly lower inorganic content when compared to enamel (96% versus 65–70% mineral content) [11]. The present study indicated that PMMA surfaces tend to have a greater wear rate than dentin surfaces (11x more) upon high duration chewing cycles mimicking a bruxism scenario. Thus, PMMA occlusal splints appear to be effective in protecting dentin-exposed teeth from further wear when considering the wear rate of the splint material. The difference in wear rate may be attributed to the mechanical properties of dentin and PMMA. Previous studies have reported similar values for the hardness of dentin and PMMA (~275–300 MPa) [18, 19]. However, PMMA is expected to cause less wear to the opposing dentin as it has a lower elastic modulus (2.6 GPa) compared to dentin (18–25 GPa) [6, 18]. The lower elastic modulus of PMMA means that it is more susceptible to deformation under stress and strain, which reduces the likelihood of causing damage. In addition, the topographical images obtained in this study indicate that the surface of dentin displays more irregularities compared to PMMA. It has been demonstrated that a rough surface can exacerbate the wear behavior of the opposing contact antagonist [20].

To the best of our knowledge, only one study conducted by Osiewicz et al. [21]. has evaluated the impact of PMMA occlusal splints on the wear of opposing dentin surfaces. In their investigation, they evaluated the effect of heat-cured and chemical-cured PMMA occlusal splints on the wear of opposing tooth surfaces. Their finding indicated that the wear of enamel, dentin, and various resin composites was higher in contact with heat-cured PMMA compared to chemical-cured PMMA. Furthermore, the highest wear was observed in dentin that was in contact with heat-cured PMMA. However, it is not possible to compare the quantitative wear rate data in our study with those of previous research due to differences in the wear test settings and models used.

In this study, different numbers of loading cycles were used to mimic a range of bruxism duration cycles seen in splint users with bruxism. Though, duration of bruxing events vary greatly from patient to patient and even within the same individual. The estimated amount of occlusal force in bruxers can be approximately three times greater than those of healthy controls [22]. Night-time splint users may perform 30,000 and 60,000 loading cycles, which are comparable to the maximum total number of bruxism cycles experienced during 2 and 4 weeks, respectively [13, 22]. In comparing wear rates at different durations cycles, the wear rate on PMMA surfaces was higher with high duration cycle, while dentin surfaces displayed a marginal decrease in wear. This finding was consistent with the ultrastructure analysis that revealed more abundant abrasion lines on PMMA surfaces with high duration bruxism cycles when comparing with low duration cycles. Those topographical changes were not so obvious in dentin surfaces, which, as expected, displayed fewer smoother surfaces than PMMA occlusal splints since these latter went through sequential polishing steps during its fabrication technique.

There are specific limitations to be considered in this study. Firstly, an ideal chewing simulator does not exist in the market, perhaps due to the lack of research studies focused on wear rates upon occlusal forces. Only three-body wear assessment equipment has been recently made available for simulating low and high duration bruxism cycles, and such testing instrument was utilized in this study. This instrument employs vertical and lateral movements to simulate chewing cycles of bruxers and several parameters can be set to resemble oral environment conditions [13, 23]. Secondly, our specimens were tested in distilled water which is a different environmental factor when compared with human saliva and its intrinsic buffering conditions. Although, a previous study found that no differences in tooth surface friction exist between distilled water and human saliva [24]. Nevertheless, this bench chewing simulator was mainly used for investigating basic wear mechanisms not under the influence of saliva buffering capacities. Though, in reality, wear mechanisms are more complex as per its multifactorial etiology. For example, tooth wear events depend on loading force, mechanical properties of the material, oral environment and the opposing type of material [12, 13]. Thus, different chewing and environmental settings will be tested in future studies to better understand the effects of each individual factor and potential confounders (e.g., diet, pH) in dentin wear rates with opposing PMMA occlusal splints.

To date, there are variety of commercial materials besides PMMA that are available for fabricating occlusal splints including polyamide resin, ethylene vinyl acetate, polycarbonate, urethane dimethacrylate. Previous studies have reported different wear resistance patterns with different occlusal splint materials [14, 25, 26]. In addition, fabrication methods have also shown to produce an impact on the wear resistance of occlusal splint materials. For example, when comparing conventional and computer-aided design together with computer-assisted manufacturing (CAD/CAM) systems, CAD/CAM-based fabrication of PMMA occlusal splints exhibited a lower wear rate than conventionally produced PMMA splints [25, 27]. However, CAD/CAM fabrication may increase cost if outsourced to an external party. Although there are substantial studies on the type of materials, fabrication methods, and type of antagonist, further investigations are still necessary for evaluating an optimal occlusal splint for patients with bruxism. One of the crucial aspects that need to be considered is cost-effectiveness.

## Conclusions

The present study unveiled that PMMA occlusal splint surfaces tend to have a greater wear rate than dentin surfaces upon high duration chewing cycles mimicking bruxism events. This finding confirms that PMMA occlusal splints are effective in protecting dentin-exposed teeth from further wear. Therefore, it is still clinically reasonable to advocate for a single-arch PMMA occlusal splint therapy (either in the maxillary or in the mandibular arch) to protect opposing dentin tooth surfaces in bruxing patients.

## Data Availability

The data supporting the findings of this study are available upon request. Interested researchers may contact Prangtip Potewiratnanond, DDS, M.Sc, PhD, at the Department of Occlusion, Faculty of Dentistry, Chulalongkorn University, Thailand. Our contact information is as follows: Department of Occlusion, Faculty of Dentistry, Chulalongkorn University, 34 Henri-Dunant Rd., Pathumwan, Bangkok 10330, Thailand. Please feel free to reach out to us at prangtip.p@chula.ac.th to request access to the data.
